# A comparison of quality of life between older adults living in high and low altitude areas

**DOI:** 10.3389/fpubh.2023.1184967

**Published:** 2023-11-20

**Authors:** Shou Liu, Fei Wang, Sha Sha, Hong Cai, Chee H. Ng, Yuan Feng, Yu-Tao Xiang

**Affiliations:** ^1^Department of Public Health, Medical College, Qinghai University, Xining, Qinghai, China; ^2^Guangdong Mental Health Center, Guangdong Provincial People's Hospital (Guangdong Academy of Medical Sciences), Southern Medical University, Guangzhou, Guangdong, China; ^3^Beijing Key Laboratory of Mental Disorders, The National Clinical Research Center for Mental Disorders, Beijing Anding Hospital, The Advanced Innovation Center for Human Brain Protection, Capital Medical University, Beijing, China; ^4^Unit of Psychiatry, Department of Public Health and Medicinal Administration, Institute of Translational Medicine, Faculty of Health Sciences, University of Macau, Macau, China; ^5^Centre for Cognitive and Brain Sciences, University of Macau, Macau, China; ^6^Department of Psychiatry, The Melbourne Clinic and St Vincent's Hospital, University of Melbourne, Richmond, VIC, Australia

**Keywords:** older adults, high altitude, depression, quality of life, low altitude

## Abstract

**Background:**

High altitude is known to have a significant impact on human physiology and health, therefore, understanding its relationship with quality of life is an important research area. This study compared the quality of life (QOL) in older adults living in high and low altitude areas, and examined the independent correlates of QOL in those living in a high altitude area.

**Methods:**

Older adults living in three public nursing homes in Xining (high altitude area) and one public nursing home in Guangzhou (low altitude area) were recruited. The WHOQOL-BREF was used to measure the QOL.

**Results:**

644 older adults (male: 39.1%) were included, with 207 living in high altitude and 437 living in low altitude areas. After controlling for the covariates, older adults living in the high altitude area had higher QOL in terms of physical (*P* = 0.035) and social domains (*P* = 0.002), but had lower QOL in psychological (*P* = 0.009) domain compared to their counterparts living in the low altitude area. For older adults living in the high altitude area, smoking status was associated with higher social QOL (*P* = 0.021), good financial status was associated with higher physical QOL (*P* = 0.035), and fair or good health status was associated with higher physical (*p* < 0.001) and psychological QOL (*P* = 0.046), while more severe depressive symptoms were associated with lower QOL.

**Conclusion:**

Appropriate interventions and support to improve depressive symptoms and both financial and health status should be developed for older adults living in high altitude areas to improve their QOL.

## Introduction

Quality of life (QOL) is a widely used health outcome with multiple dimensions that involve subjective sense of mental and physical status, social roles and functioning, personal relationships, and environmental factors (1). Among different age groups, QOL in older adults is a topic of concern (2). During the past decade, the research on QOL in older adults has mainly focused on its relationships with impairments of body function (3), physical and mental illnesses (4), cognitive deficits (5), and increased social and economic burden (6).

Previous studies found that unhealthy lifestyle factors (e.g., heavy smoking and drinking) were associated with lowered QOL (7–10) that was moderated by impaired physical (11, 12) and mental health status (13, 14). In addition, cognitive impairment was common among older adults living in high altitude areas, which was not associated with QOL in this population (5). In contrast, the influence of certain environmental factors on QOL is relatively less well-understood or studied. Living at high altitude has well-known impacts on health status due to hypobaric hypoxia in high-altitude areas for both local residents and those who recently arrived (15, 16). Some studies found that genetic factors in long-term high altitude residents could play a role in adapting to their local environment better than those living in other areas (17–21). Further, other studies examined the direct impact of high altitude on health including the influence of living in moderate altitude areas on the reproductive function (22), metabolic syndrome (23), and other cardiometabolic functions (24). Overall, acute mountain sickness is the most common discomfort experienced by newcomers to high altitude areas, which is associated with sleep problems and headache, dizziness, nausea and vomiting, sleep disturbance and fatigue (15). Chronic mountain sickness (CMS) that affects local residents in high altitude areas is related to a progressive loss of ventilatory rate, which naturally occurs with age and result in excessive hypoxemia and polycythemia (25). Some studies found that residents living in high altitude areas were more likely to suffer from high-altitude polycythemia (26), high-altitude pulmonary hypertension (27), congenital cardiac anomalies (28, 29), chronic kidney (30), and rheumatoid arthritis (31) due to hypoxia compared to those living in low altitude areas, all of which are negatively associated with QOL.

On the other hand, moderate altitude levels could have positive effects on sugar metabolism and blood pressure. For example, inhabitants living in the Valley of Mexico (2,240 m above sea level) were found to breathe 29% more on average and have 10% higher hemoglobin concentrations compared to residents living near sea level. Young adults in Mexico City had an SaO2 between 92 and 94% vs. 97% among those living at sea level (32). It is likely that lung growth, development during pregnancy and infancy, and other physiological adaptations are enhanced in moderate altitude levels, although solid evidence is still needed. However, for people with respiratory diseases, residing at moderate altitudes could result in worsening hypoxemia and clinical deterioration (32).

To date there have been no comparative studies on the influence of living in high altitude areas on QOL in older adults. This study hence compared the QOL in older adults living in high and low altitude areas and explored the correlates of QOL in those living in high altitude area.

## Methods

### Study design and participants

This was part of a cross-sectional, comparative study on mental health in older adults living in high and low altitude areas (33). The surveys were conducted from September 1st to November 31st in 2019 in three public nursing homes in Xining that is the capital city of Qinghai province with an average altitude of 2,300 m (high altitude area), and one public nursing home in Guangzhou which is the capital city of Guangdong province with an average altitude of 10 m (low altitude area). Older adults included in this study were living in the selected nursing homes, aged 60 years or above and able to understand the purpose and content of the assessment. Those with severe cognitive problems (e.g., dementia and severe head injury) as determined by a review of health records were excluded. The study protocol was approved by the Ethical Committee of the University of Macau. Written informed consent were provided by all participants.

### Instruments

Basic sociodemographic and clinical characteristics, such as age, gender, marital status, education level, perceived financial and health status, and having a religion and major medical conditions, were collected in an interview and confirmed by a review of health records. QOL was measured using the validated Chinese version of the WHO Quality of Life brief version–WHOQOL-BREF (34, 35) that consists of 26 items covering four domains: physical, psychological, social relationships, and environment. Each item were scored from 1 to 5 points (36), with a higher score indicating higher QOL.

The validated Chinese version of the Patient Health Questionnaire (PHQ-9) (37, 38) was used to measure the severity of depression. The PHQ-9 consists of nine items with each scored from 0 = “not at all”, 1 = “several days” to 2 = “more than half of the days” and 3 = “nearly every day”. The PHQ-9 total score ranges from 0 to 27, with a higher score indicating more severe depression.

### Data analyses

The sociodemographic and clinical characteristics of older adults living in the high and low altitude areas were compared using chi-square test, two independent samples *t*-test and Mann-Whitney *U-*test as appropriate. Then QOL between older adults living in the high and low altitude areas was compared using analysis of covariance (ANCOVA) after controlling for those that significantly differed in univariate analyses. The associations between socio-demographic and clinical characteristics and QOL in older adults living in high altitude area were examined using two independent samples *t-*test, analysis of variance, chi-square test, and Pearson correlation analysis. Finally, multiple linear regression analyses were used to analyze the independent correlates of QOL in older adults living in the high altitude area. Each of the physical, psychological, social relationships, and environmental QOL was the dependent variable, while demographic and clinical variables that significantly differed in univariate analyses were entered as the independent variables. IBM SPSS Statistics for Windows, version 24.0 (IBMCorp., Armonk, N.Y., USA) was used to analyze data. *P-*value was set at 0.05 (two-tailed).

## Results

Altogether, 644 older adults were included, with 207 in high and 437 in low altitude areas who completed the assessment. There were significant differences between older adults living high and low altitude areas in terms of age, education, religion, perception of financial status, perception of health status, number of major medical conditions, psychological QOL and PHQ-9 total score (Table 1). ANCOVA revealed that after controlling for variables that significantly differed in the univariate analyses, older adults living in high altitude area had significantly higher physical [*F*_(1, 644)_ = 4.46, *P* = 0.035] and social QOL [*F*_(1, 644)_ = 9.30, *P* = 0.002], and lower psychological QOL [*F*_(1, 644)_ = 6.94, *P* = 0.009] compared to those living in low altitude area. There was no group difference in environmental [*F*_(1, 644)_ = 0.32, *P* = 0.570] QOL.

**Table 1:** Basic demographic and clinical characteristics of the whole sample.

	**The whole sample (*****n*** = **644)**		**Low altitude (*****n*** = **437)**		**High altitude (*****n*** = **207)**		**Statistics**		
	***N***	**%**	***N***	**%**	***N***	**%**	**X^2^**	***df* ^a^**	***P***
**Gender**							3.62	1	0.057
Female	**392**	**60.9**	**277**	**63.4**	**115**	**55.6**			
Male	252	39.1	160	36.6	92	44.4			
Married/cohabitating	136	21.1	87	19.9	49	23.7	1.19	1	0.275
Secondary school or above	274	42.6	198	45.3	76	36.7	4.24	1	**0.039**
Smoking	136	21.1	91	20.8	45	21.7	0.07	1	0.790
Having a religion	504	78.3	423	96.8	81	39.1	321.90		**< 0.001**
**Perception of financial status**							31.31	2	**< 0.001**
Good	256	39.8	144	32.9	112	54.1			
Fair	290	45.0	210	48.0	80	38.6			
Poor	98	15.2	83	19.0	15	7.2			
**Perception of health status**							24.62	2	**< 0.001**
Good	154	23.9	84	19.2	70	33.8			
Fair	374	58.1	282	64.5	92	44.4			
Poor	116	18.0	71	16.2	45	21.7			
Presence of major medical conditions^d^	609	94.56	430	98.40	179	86.47	38.86	1	**< 0.001**
	**Mean**	**SD**	**Mean**	**SD**	**Mean**	**SD**	**T/Z**	*df* ^b^	***p***
Age (years)	80.4	8.6	81.5	8.3	78.12	8.8	4.65	642	**< 0.001**
PHQ-9 total	3.0	3.9	1.6	2.8	6.02	4.1	−14.83	–^**c**^	**< 0.001**
Physical QOL	13.2	2.0	13.1	1.8	13.2	2.3	0.385	642	0.700
Psychological QOL	13.5	2.1	13.6	1.9	13.3	2.3	2.061	642	**0.040**
Social QOL	13.4	1.8	13.4	1.5	13.6	2.4	1.210	642	0.227
Environmental QOL	13.4	1.9	13.4	1.8	13.2	2.2	1.052	642	0.293

*df* , degree of freedom; SD, standard deviation; PHQ-9, Patient Health Questionnaire-9; QOL, quality of life.

^a^χ^2^ test.

^b^Two sample independent *t*-test.

^c^Mann-Whitney *U*-test.

^d^Major medical conditions including included Hypertension, Cardiopathy, Stroke, Parkinson, Diabetes, Asthma, Chronic bronchitis Emphysema, Pulmonary disease, Liver disease, Nephropathy, Thyroid dysfunction, Arthritis, Cancer, racture/Osteoporosis/Humpback, Other diseases.

Bolded values: *p* < 0.05.

Table 2 shows the associations between basic and clinical demographic characteristics and QOL domains in older adults living in high altitude areas. Male gender was significantly associated with physical and psychological QOL, while smoking status, perceived health status and depressive symptoms were associated with all four QOL domains. Perceived financial status, medical conditions and age were also associated with physical QOL.

**Table 2:** Associations between socio-demographic characteristics and QOL domains in older adults in high altitude region.

	**Physical QOL**			**Psychological QOL**			**Social QOL**			**Environmental QOL**		
	**Mean** ±**SD**	**T/F**	***P***	**Mean** ±**SD**	**T/F**	**P**	**Mean** ±**SD**	**T/F**	***P***	**Mean** ±**SD**	**T/F**	***P***
**Gender**		2.20	**0.029**		2.18	0.030		0.59	0.556		0.64	0.522
Male	13.6 ± 2.5			13.7 ± 2.5			13.7 ± 2.5			13.4 ± 2.4		
Female	12.9 ± 2.0			13.0 ± 2.0			13.5 ± 2.4			13.2 ± 2.1		
**Marital status**		0.92	0.357		1.42	0.157		0.40	0.689		0.47	0.636
Single/devoiced/widow	13.1 ± 2.2			13.1 ± 2.2			13.5 ± 2.2			13.3 ± 2.1		
Married/cohabitating	13.4 ± 2.5			13.7 ± 2.5			13.7 ± 3.0			13.1 ± 2.6		
**Education**		0.24	0.807		0.77	0.444		0.75	0.455		0.47	0.637
Illiterate or primary school	13.1 ± 2.2			13.2 ± 2.2			13.5 ± 2.3			13.3 ± 2.0		
Secondary school or above	13.2 ± 2.5			13.4 ± 2.3			13.7 ± 2.6			13.1 ± 2.5		
**Smoking**		2.29	**0.023**		2.57	**0.011**		2.94	**0.004**		2.04	**0.043**
No	13.0 ± 2.1			13.1 ± 2.3			13.3 ± 2.3			13.1 ± 2.1		
Yes	13.9 ± 2.7			14.0 ± 2.1			14.5 ± 2.5			13.8 ± 2.4		
**Having a religion**		0.19	0.845		0.98	0.327		0.43	0.668		1.58	0.114
No	13.2 ± 2.2			13.4 ± 2.2			13.6 ± 2.3			13.4 ± 2.1		
Yes	13.1 ± 2.4			13.1 ± 2.3			13.5 ± 2.6			12.9 ± 2.3		
**Perception of financial status**		6.13	**0.003**		2.78	0.064		2.95	0.055		1.59	0.207
Good	13.7 ± 2.3^ab^			13.6 ± 2.4^b^			13.9 ± 2.4^b^			13.5 ± 2.5		
Fair	12.7 ± 2.1			12.8 ± 2.0			13.1 ± 2.2			12.9 ± 1.8		
Poor	12.0 ± 2.4			13.3 ± 1.3			13.5 ± 3.0			13.0 ± 1.7		
**Perception of health status**		32.89	**< 0.001**		4.02	**0.019**		3.99	**0.020**		3.35	**0.037**
Good	14.6 ± 2.1^ab^			13.7 ± 2.4^a^			14.2 ± 2.5^ab^			13.7 ± 2.4^a^		
Fair	13.0 ± 2.0^a^			13.3 ± 2.2			13.4 ± 2.3			13.2 ± 2.1		
Poor	11.5 ± 1.7			12.5 ± 1.9			13.0 ± 2.4			12.6 ± 2.1		
**Presence of major medical conditions**		3.02	**0.003**		0.95	0.344		1.24	0.218		1.03	0.308
Good	14.4 ± 2.5			13.6 ± 2.2			14.1 ± 2.5			13.7 ± 2.4		
Poor	13.0 ± 2.2			13.2 ± 2.2			13.5 ± 2.4			13.2 ± 2.2		
	***r**_*p*_*		***P***	***r**_*p*_*		***P***	***r**_*p*_*		***P***	***r**_*p*_*		***P***
Age (years)	−0.17		**0.013**	−0.04		0.528	−0.01		0.910	−0.07		0.302
PHQ-9 total	−0.32		**< 0.001**	−0.33		**< 0.001**	−0.30		**< 0.001**	−0.27		**< 0.001**

SD, standard deviation; PHQ-9, Patient Health Questionnaire-9; QOL, quality of life; r_p_, Pearson's Correlation coefficient; T, two sample independent *t*-test; F, analysis of variance.

^a^Compare with *Poor* group, *p* < 0.05.

^b^Compare with *Fair* group, *p* < 0.05.

Bolded values: *p* < 0.05.

Table 3 presents the results of multiple linear regression analyses in older adults living in high altitude areas. For older adults living in high altitude area, smoking status was associated with higher social QOL (β = 0.16, 95% CI: 0.14–1.74, *P* = 0.021), while good financial status was associated with higher physical QOL (β = 0.24, 95% CI: 0.08–2.16, *P* = 0.035), fair or good health status were associated with higher physical QOL (fair health status: β = 0.30, 95%CI: 0.71–2.09, *p* < 0.001; good health status: β = 0.54, 95%CI: 1.89–3.39, *p* < 0.001) and psychological QOL (β = 0.18, 95% CI: 0.02–1.68, *P* = 0.046), and more severe depressive symptoms were associated with lower physical (β = −0.19, 95% CI: −0.17 to −0.04, *P* = 0.002), psychological (β = −0.29, 95% CI: −0.23 to −0.08, *P* < 0.001), social (β = −0.26, 95% CI: −0.24 to −0.07, *P* < 0.001), and environmental QOL (β = −0.23, 95% CI: −0.20 to −0.05, *P* = 0.001).

**Table 3:** Socio-demographic correlates of QOL in older adults living in high altitude region (by multiple linear regression analysis).

	**Physical QOL**			**Psychological QOL**			**Social QOL**			**Environmental QOL**		
	***P***	β	**95%CI**	***P***	β	**95%CI**	***P***	β	**95%CI**	***P***	β	**95%CI**
Male gender	0.472	0.04	−0.36 to 0.77	0.211	0.09	−0.23 to 1.02	0.584	−0.04	−0.86 to 0.49	0.665	−0.03	−0.77 to 0.50
Smoking	0.231	0.07	−0.03 to 1.07	0.082	0.12	−0.08 to 1.40	**0.021**	0.16	0.14–1.74	0.218	0.09	−0.28 to 1.23
**Perception of financial status**												
Fair	0.556	0.07	−0.75 to 1.39	0.182	−0.17	−1.99 to 0.38	0.166	−0.18	−2.19 to 0.38	0.510	−0.09	−1.61 to 0.80
Good	**0.035**	0.24	0.08–2.16	0.808	−0.03	−1.30 to 1.02	0.902	−0.02	−1.33 to 1.17	0.776	0.04	−1.01 to 1.35
**Perception of health status**												
Fair	**< 0.001**	0.30	0.71–2.09	0.112	0.14	−0.15 to 1.38	0.490	0.06	−0.54 to 1.12	0.222	0.11	−0.30 to 1.26
good	**< 0.001**	0.54	1.89–3.39	**0.046**	0.18	0.02–1.68	0.071	0.16	−0.07 to 1.73	0.089	0.16	−0.11 to 1.59
Presence of poor major medical conditions	0.421	−0.05	−1.12 to 0.47	0.984	0.01	−0.88 to 0.90	0.673	−0.03	−1.16 to 0.75	0.759	−0.02	−1.04 to 0.76
Age (years)	0.109	−0.10	−0.06 to 0.01	0.346	0.07	−0.02 to 0.05	0.276	0.08	−0.02to 0.06	0.854	−0.01	−0.04 to 0.03
PHQ-9 total	**0.002**	−0.19	−0.17 to −0.04	**< 0.001**	−0.29	−0.23 to −0.08	**< 0.001**	−0.26	−0.24 to −0.07	**0.001**	−0.23	−0.20 to −0.05

CI, confidence interval; PHQ-9, Patient Health Questionnaire-9; QOL, quality of life.

β refer to standard coefficient of multiple linear regression analysis.

Bolded values: *p* < 0.05.

## Discussion

This was the first comparative study of older adults that examined the association between living at high altitude areas and QOL. We found that older adults living in high altitude area had higher physical and social QOL, but lower psychological QOL compared to their counterparts living in low altitude area. For older adults living in high altitude area, smoking status, perceived financial and health status, and depressive symptoms were associated with QOL.

Older adults living in high altitude area had lower psychological QOL than those living in low altitude area, which could be due to increased risk of psychological and psychiatric sequelae of residing in high altitude areas, such as sleep disturbances (33, 39), stress, depression, anxiety (40) and even suicide (41). This was partly supported by our study findings in that those with more severe depressive symptoms were more likely to have lower QOL. Moreover, high psychological stress could also contribute to poor physical health such as heart disease (42) and cancer (43), which in turn could further lower psychological QOL.

There are several possible reasons for the association between high altitude and increased risk of psychiatric symptoms. Living at high altitude is associated with hypobaric hypoxia, which could impair brain functions over time (44) and increase the likelihood of psychiatric comorbidities including depression (40). In addition, persons living at high altitude are exposed to chronic hypoxia, which could lead to a number of physical diseases, such as high-altitude polycythemia (26), high-altitude pulmonary hypertension (27), and congenital cardiac anomalies (28, 29). All these factors could increase the risk of mental health problems due to the respective burden of the disease and treatment which could lower QOL.

Unexpectedly, our study revealed that adults living in a high altitude area had higher QOL in both physical and social domains but no difference in environmental domain compared to their counterparts living in low altitude area. This could be explained by the following reasons. QOL is largely determined by the gap between one's expectation and actual experiences (45). In the past decade, the central Chinese government has allocated substantial budget and health resources to improve the health service systems and living conditions for the population living in Qinghai-Tibet Plateau, particularly cities such as Xining city where this study was conducted. Therefore, the participants' experiences in terms of physical, social and environmental aspects would be expected to improve, which would match or even exceed their earlier expectations. However, like most areas across China, the implementation of mental health promotion and services (e.g., the Psychological Care Project for Older Adults) were still under-developed in the community which might influence psychological QOL. In addition, environmental and social factors could play an important role on QOL (46). In developed areas such as in Guangzhou (low altitude area), crowded living, noise pollution, fast working pace, competitive pressure and stress of high living cost could negatively influence QOL; in contrast, these factors were less evident in economically under-developed area such as Xining (high altitude area), which might partly explain the finding that adults living in high altitude area had higher QOL in both physical and social domains.

Several sociodemographic and clinical characteristics were associated with QOL in this study. Smoking could increase the risk of a range of physical diseases (47–49), which in turn could lowered QOL (50, 51). However, surprisingly older adults who smoke had higher social QOL in this study. In traditional Chinese culture, smoking and drinking are common social behaviors used to enhance social networks, facilitate interpersonal interactions, increase social activity of older people to reduce loneliness (52–54), all of which could improve QOL. Consequently, compared to those who do not smoke, older adults who smoke usually have a better social network and supports (55, 56), which could improve QOL in the social domain. As expected, older adults with better perceived financial and health status were more likely to have higher QOL. Better financial situation and health status are usually associated with greater level of health literacy and access to healthcare services (57, 58) as well as good social support (59) in older adults, all of which could improve QOL.

There are several limitations in this study. First, due to the cross-sectional study design, causal relationship between QOL and other variables could not be examined. Second, for logistical reasons, the participants were invited from nursing homes and those with obvious cognitive problems were excluded, which would limit the generalizability of the findings. Finally, some variables related to QOL, such as treatment of physical diseases, were not examined.

In conclusion, our study of older adults found a significant association between living at high altitude and QOL. Older adults living in a high altitude area had higher physical and social QOL, but lower psychological QOL. Appropriate interventions to address depressive symptoms (e.g., increase access to community mental health services), and support to improve financial and health status should be developed for older adults living in high altitude areas to improve their QOL.

## Data availability statement

The datasets presented in this article are not readily available because the Clinical Research Ethics Committee of University of Macau that approved the study prohibits the authors from making publicly available the research dataset of clinical studies. Requests to access the datasets should be directed to xyutly@gmail.com.

## Ethics statement

The studies involving human participants were reviewed and approved by the Ethical Committee of the University of Macau. The patients/participants provided their written informed consent to participate in this study.

## Author contributions

YF and Y-TX: study design. SL, FW, SS, HC, and Y-TX: collection, analyses, and interpretation of data. SL and Y-TX: drafting of the manuscript. CN: critical revision of the manuscript. All authors contributed to the article and approved the submitted version.
